# The activity of trypsin in the pancreatic juice and blood of poultry increases simultaneously in the postprandial period

**DOI:** 10.3389/fphys.2022.874664

**Published:** 2022-10-25

**Authors:** Vladimir G. Vertiprakhov, Natalya V. Ovchinnikova

**Affiliations:** ^1^ Timiryazev Russian State Agrarian University Moscow Agrarian Academy, Moscow, Russia; ^2^ Physiology of Motivation Laboratory, Anokhin Research Institute of Normal Physiology, Moscow, Russia

**Keywords:** trypsin, pancreatic juice, broiler chickens, adaptation to diet composition, postprandial secretion

## Abstract

Modern literature data indicate that the role of trypsin goes far beyond its digestive function. Once in the blood, trypsin is involved as part of the kallikrein-kinin system in the regulation of blood pressure, regulates pancreatic function by activating PAR receptors, and influences inflammation and immunity in the cell. The interaction of trypsin in the intestine and serum in the living healthy organism has been insufficiently studied. On the basis of our own studies and literature data, we concluded that after overnight fasting the increase of trypsin activity in pancreatic juice and blood serum in the postprandial period occurs in parallel, which determines not only digestion of food protein but also the level of metabolism. Consequently, determining the optimal amount of crude protein in the diet during the morning meal is a paramount task for physiologists.

## Introduction

Trypsin is secreted by the pancreas as trypsinogen, is activated by enterokinase in the duodenum, and hydrolyzes protein molecules to peptides and amino acids. Trypsin is an optimal marker for detecting changes in the physiological state of the pancreas because it is specific for this organ. It has been established that trypsin administration into the blood decreases enzymes output with pancreatic juice, and on the contrary, trypsin inhibitor administration is accompanied by enzymes secretion increase (Sheida et al., 2022).

The role of trypsin goes far beyond its digestive function. It was found that trypsin is involved in the activation of kallikrein, which regulates blood pressure (Chia et al., 2011). In addition, trypsin is an activator of PAR-receptors, which provide transmission of information to the cell during inflammatory processes and immunological reactions (Yarovaya et al., 2009). PAR2 activators have been shown to affect the pancreas and regulate the secretory function of the pancreas, stomach and salivary glands. There is evidence that PAR receptors activated directly by trypsin indicate a role for the enzyme in the pathogenesis of neurodegenerative diseases of the brain (Luo et al., 2007; Wang et al., 2008; Lohman et al., 2009; Almonte et al., 2013). Unlike other members of the PAR family, which are effectively activated by thrombin, PAR2 represents a class of receptors activated by trypsin (Nieman, 2016).

Knowledge of the mechanisms of trypsin involvement in the norm and in the development of pathological processes is relevant because of pancreatic diseases, as there is a growing trend in the frequency of this disease worldwide (Forsmark, 2013; De la Iglesia-Garcia et al., 2018; Zhang et al., 2018). Knowledge of the interaction between trypsin-like enzymes and alpha-1-proteinase inhibitor (α1-PI) is particularly relevant because the role of α1-PI in regulating viral tropism to human cells has been established. Patients with genetic deficiency of α1-PI show increased susceptibility to SARS-CoV-2 virus and their infection has the most unfavorable form (Akbasheva et al., 2022).

Based on the literature and our own data, we showed pancreatic regulation during food intake and the role of trypsin as a trigger mechanism in metabolic regulation by examining trypsin activity in the duodenum and serum in poultry during the postprandial period.

## Opinion is pancreatic secretion parallel or non-parallel?

In the world gastroenterological literature, the intensity of discussions about the expedient, adaptive secretion of the major digestive glands has not faded for more than a century. From our point of view, academician I.P. Pavlov’s idea of the adaptive secretion of digestive enzymes, born in his laboratory the century before last, has lost its relevance in its original formulation and new research using modern approaches are required. For more than 70 years, it has been persistently studied exclusively at the organ level.

Most researchers adhere to the idea of I.P. Pavlov that pancreatic enzymes are secreted based on diet composition (Pavlov, 1951). The results of research in the second half of the 20th century supplemented and developed the theory of adaptation of pancreatic secretion to diet composition (Ugolev, 1961; Klimov and Fokina, 1987; Case, 1998; Korotko, 2005).

The situation changed radically after the results obtained by Palade, (1975) in the study of the processes of protein biosynthesis in the pancreatic cell. This work was awarded the Nobel Prize in 1974 (Palade, 1975). The authors found that zymogen granules contain all the enzymes produced by the gland. It was shown that all acinar cells of the pancreas are homogeneous, all of them, according to the same mechanisms of biosynthesis of any protein molecules, synthesize in one ratio the entire set of enzymes intended for export. In the process of ribosomal synthesis and subsequent intracellular transport, all these enzymes are completed, packed into separate secretory granules, and appear in the excretory pancreatic duct in this form. Each secretory granule has a strictly constant (depending on the type of animal and its age), unchanged composition in terms of the quantitative composition of all enzymes exported by the cell. The existing mechanisms that function according to the laws of biochemical logic do not leave the slightest possibility of influencing both the intensity of the biosynthesis of individual digestive enzymes and the rate of their secretion. Therefore, in contrast to Pavlov’s adaptive, expedient theory of biosynthesis and secretion, Palade’s hypothesis was called the theory of parallel secretion, i.e. strictly linked simultaneous and identical secretion of all enzymes synthesized and intended for export to the digestive tract.

At the same time, in the 70s of the last century, Russian scientists (Permyakovet al., 1973) established ultrastructural changes in acinar cells for each diet. With mixed or protein diet, changes in cell ultrastructures are similar and unfold cyclically in the cellular conveyor in full accordance with the biorhythm of acinar cells. A fat diet causes a restructuring of the acinar cell, affecting all its elements. There is hyperplasia and increased degranulation of the rough endoplasmic reticulum; transformation of rough vacuoles into smooth ones; hypertrophy of mitochondria and the Golgi complex. A carbohydrate diet is characterized by significant vacuolization of the endoplasmic reticulum with its subsequent reduction; decreased activity of the Golgi complex, mitochondrial hypertrophy. The physiological meaning of this structural rearrangement is the adaptation of the acinar cell to the predominant production of enzymes necessary for the processing of this type of food: lipases - with a fat diet, amylases - with a carbohydrate diet, proteases–with a protein diet (Di Magno and Layer, 1993; Case, 1998; Chandra and Liddle, 2015). It is the universality of the cellular “conveyor” of the acinar cell, adapted to the simultaneous synthesis of many enzymes, that allows it to be restructured when changing the diet. In addition, it was shown that there are peculiar bipolar islet-acinar cells in the pancreas, one-half of which, oriented towards the centroacinous duct, synthesizes zymogen granules, the other half, in close contact with the capillary network, produces granules similar in size, shape and degree of osmiophilia to those in endocrine cells. The emerging data on the presence of pancreatic enzymes in the blood have expanded the understanding of proteases role in metabolic processes in the body.

## How enzymes are formed in the pancreas

It is known that the exosecretions of the digestive glands contain two pools of enzymes: newly synthesized and recreated. The rate of enzyme synthesis does not keep pace with exosecretion, which was shown when the enzyme secreting activity of the pancreas was taken into account (Rothman et al., 2002). Therefore, enzyme synthesis deficiency is compensated by their recreation. There are three ways enzymes enter the blood: endosecretion is the first way of transporting enzymes from glandulocytes to the interstitium, and then from it to the lymph and the bloodstream. The second way of transporting enzymes into the bloodstream is the enzymes resorption from the ducts of digestive glands (salivary, pancreatic and gastric) - “avoidance” of enzymes and the third way–enzymes enter the blood in the small intestine, mainly in the ileum (Korotko, 2006, 2007, 2011). Enzyme homeostasis is maintained by renal and extrarenal excretion of enzymes from the body, enzymes degradation by serine proteinases, and enzymes inactivation by specific inhibitors. The latter is relevant to serine proteinases - trypsin and chymotrypsin. Their main inhibitors in blood plasma are α1-proteinase inhibitor and α2-macroglobulin. The former completely inactivates pancreatic proteinases, while the latter only limits their ability to break down high molecular weight proteins (Gőtze and Rothman, 1975; Liebow and Rothman, 1975; Heinrich et al., 1979; Korotko et al., 1986; Dosenko et al., 1999; Veremeenko et al., 2000, 2005). This complex has substrate specificity only for some low molecular weight proteins. It is not sensitive to other inhibitors of blood plasma proteinases, does not undergo autolysis, does not show any antigenic properties, but is recognized by cell receptors, and causes the formation of physiologically active substances in some cells (Dosenko et al., 1999).

Enzymes enter glandulocytes from the bloodstream, where they were transported by endosecretion, resorption from ducts reservoirs and small intestine, where they have a stimulating or inhibitory effect on enzymes secretion and, where they are recreated by glandulocytes together with their “own” enzymes. At this level of the secretory cycle, the signaling role of enzymes in the formation of the final enzyme spectrum of exosecretion is implemented using the principle of negative feedback at the level of the intracellular process, which was shown in experiments *in vitro* (Rothman, 1980). This principle is also used in the self-regulation of pancreatic secretion through reflex and paracrine mechanisms. Postprandially, portions of the secret deposited in the ducts are first transported into the cavity of the digestive tract, then portions of the secret with recreated enzymes, and, finally, the secret with recreated and newly synthesized enzymes is transported.

The concept of two enzyme pools of digestive glands exosecretions removes the question of the quantitative discrepancy between secreted and urgently synthesized enzymes by the digestive glands, since exosecretions always make up the sum of these two pools of enzymes. The ratios between the pools can change in the dynamics of exosecretion due to their different mobility in the postprandial period of glandular secretion. The recretory component of exosecretion is largely determined by the transport of enzymes into the bloodstream and the content of enzymes in it, changing in normal and pathological conditions. These processes are regulated by nervous and humoral mechanisms, apparently, with the participation of trypsin.

In the laboratory of Pavlov, it was shown that when fistula dogs lose pancreatic juice, hypersecretion of the gland is observed at first, and intraduodenal administration of juice inhibits its secretion. This reverse or recurrent inhibition of secretion has been confirmed in experiments on rats, guinea pigs, cats, dogs, productive animals and humans (Korotko, 1992; Korotko and Voskanyan, 2001, 2005; Liddle, 2006). It was found that the effect of inhibition of pancreatic secretion is caused by intraduodenal introduction of proteases (trypsin, chymotrypsin and their zymogens), and this inhibition is removed by a trypsin inhibitor (Korotko and Baibekova, 1989). The specificity and high selectivity of the reverse inhibition of trypsin secretion was demonstrated in the experiments when an artificial trypsin-specific substrate, BAPNA (benzoyl-arginine-p-nitroanilide), was introduced into the duodenum, and it increased the activity of only trypsin (Korotko and Baibekova, 1989).

**FIGURE 1:**
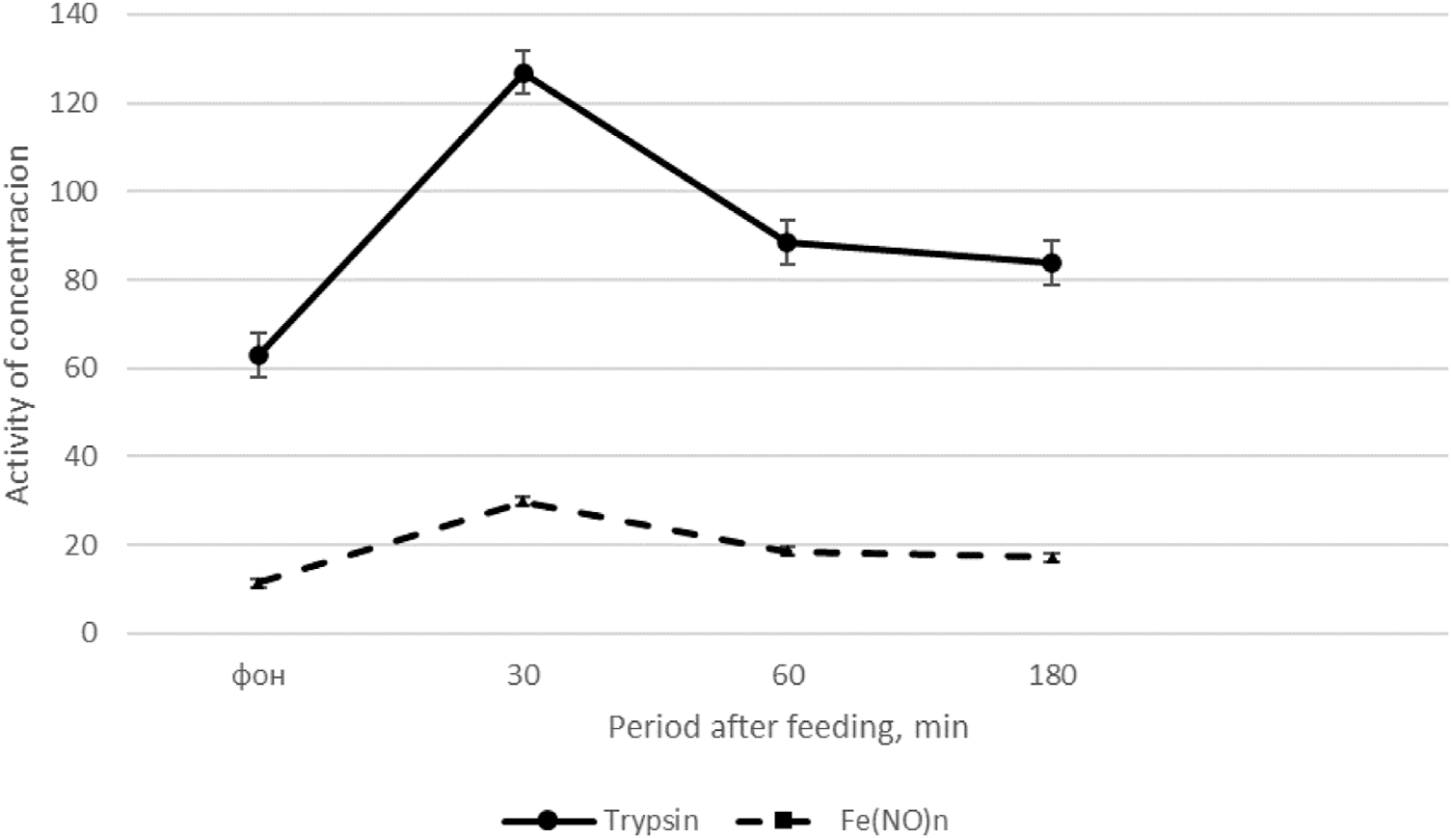
Dynamics of trypsin activity and the content of Fe(NO)n in cockerels after feeding (Fisinin V. I. et al., 2018).

The results of the study on cockerels (Fisinin V. I. et al., 2018), presented in Figure 1 show that feed intake after a 14-h fast causes the most significant changes in trypsin activity. The activity of amylase and lipase in the blood plasma after feed intake does not change significantly. Moreover, the interval after feed intake is important: the greatest increase in trypsin activity in the blood is observed 30 min after feeding (by 66.0–67.1%) compared with the basal level of activity. 180 min after feeding trypsin activity in the blood decreases by 24.8% compared to trypsin activity 30 min after feed intake (see Figure 1).

On the *x*-axis - the period after feeding, min; on the *y*-axis, units of trypsin activity (U/L) and Fe(NO)n, μM/l. The solid line is the dynamics of trypsin activity, the dotted line is the content of Fe(NO)n.

Thus, feed intake significantly increases trypsin activity and the content of NO donors in the blood plasma of roosters maximum 30 min after feeding, and then the indicators gradually decrease, approaching the initial level 180 min after feed intake.

## In addition for the hypothesis, there is an inverse relationship between trypsin in the blood and pancreatic juice

The results of the experiment on poultry differ significantly from the data obtained on rats (Laporte and Tremolieres, 1971; Korotko and Baibekova, 1989), which showed an inverse relationship between trypsin in the blood and pancreatic juice. In experiments on chickens, when intravenous solutions of trypsin or pancreatic juice were administered to them, reverse inhibition and a decrease in the activity of duodenal proteases by 25.7–50.0% 30 min after injection were noted, which is apparently associated with the effect of trypsin on the control center of digestive system (medulla oblongata) (Vertiprakhov and Grozina, 2020). Therefore, an increase in trypsin activity in pancreatic juice and blood in the first 30 min after feeding can be explained by reflex effects on the secretory function of the pancreas, and subsequent regulation and adaptation to food composition is achieved by humoral factors, including participation of trypsin.

Analyzing the experimental data of pancreatic secretion in farm animals (Fisinin V. I. et al., 2018, 2019; Lebedev et al., 2019), it can be stated that in experiments with chronic pancreatic fistula, there are changes in pancreatic secretion during food intake, as well as those associated with the diet composition. The peculiar bipolar islet-acinar cells, which have a secretory and endocrine function, found during the ultrastructural analysis of the secretory cycle (Permyakov et al., 1973), suggest the possibility of simultaneous entry of trypsinogen both into the pancreatic duct and the bloodstream.

## Discussion

The flow of appetitive pancreatic juice (rich in enzymes) into the duodenum begins immediately after a meal, at the same time the activity of trypsin in blood serum increases, and the function of the whole digestive conveyor throughout the day depends on this process. The work contributes to the issues of pancreatic secretion regulation by clarifying the hypothesis of inverse dependence of pancreatic enzymes on enzymes in the blood of animals by stating that in the postprandial phase the enzyme activity increases in parallel, simultaneously regulating the processes of hydrolysis of nutrients and their assimilation. Taking into account the formation of a conditioned reflex to individual components of the diet, the maximum level of assimilation of nutrients is achieved. Consequently, the morning meal is decisive in the daily diet and therefore further research should be aimed at studying the optimal level of protein in the morning diet.

Further study of protease stimulation during the morning meal, taking into account the role of trypsin as a hormone-like substance, suggests that this direction is promising for further research.
